# Site‐specific hyperphosphorylation of tau and C‐terminal alteration of PP2A catalytic subunit in tau pathogenesis seeded by proteopathic tau

**DOI:** 10.1002/alz.094789

**Published:** 2025-01-09

**Authors:** Fei Liu, Jianlan Gu, Yan Zhou, Wen Hu, Ruozhen Wu, Yunn Chyn Tung, Cheng‐Xin Gong, Khalid Iqbal

**Affiliations:** ^1^ New York State Institute for Basic Research in Developmental Disabilities, Staten Island, NY USA

## Abstract

**Background:**

Regional distribution of neurofibrillary tangles consisting of hyperphosphorylated tau correlates strongly with the progression of Alzheimer’s disease (AD). Misfolded proteopathic tau templates the conversion of naive tau into a pathological state in a prion‐like fashion, which underlies the spreading of tau pathology in the brain. Whether hyperphosphorylation triggers tau aggregation or hyperphosphorylation occurs after aggregation is under much debate.

**Method:**

In the present study, by using oligomeric tau derived from AD brain (AD O‐tau) as proteopathic tau seeds, we determined the role and molecular mechanism of site‐specific hyperphosphorylation in seeded‐tau aggregation in cultured cells and *in vivo* in mice.

**Result:**

We found that seeded‐tau aggregation in cultured cells and *in vivo* was accompanied by site‐specific hyperphosphorylation of tau and by reduction in the level of PP2A catalytic subunit (PP2Ac) determined by a body of antibodies targeting its C‐terminus, but not its middle region. PP2Ac immunoreactivity with the C‐terminal antibodies was reversely correlated with the hyperphosphorylation of tau in cultured cells and in AD brain. Site‐specific hyperphosphorylation of tau and reduction of PP2Ac immunoreactivity were co‐existed in RIPA‐insoluble fraction and associated with seeded tau aggregation. Blocking the site‐specific hyperphosphorylation by mutating Ser/Thr to Ala suppressed the seeded‐tau aggregation and the decline of PP2Ac immunoreactivity. Knockdown and overexpression of PP2Ac enhanced and suppressed seeded‐tau aggregation in cultured cells and tau pathology *in vivo*, respectively.

**Conclusion:**

These findings strongly suggest that site‐specific hyperphosphorylation of tau is essential for seeded‐tau aggregation. Site‐specific hyperphosphorylation of tau, tau aggregation, and C‐terminal alteration of PP2Ac work synergistically to produce tau pathology. Interrupting this vicious cycle may enable inhibition of the propagation of tau pathology and the progression of AD and related tauopathies.

